# Protecting clinical trials in cystic fibrosis during the SARS-CoV-2 pandemic: risks and mitigation measures

**DOI:** 10.1186/s13063-021-05457-5

**Published:** 2021-08-28

**Authors:** Silke van Koningsbruggen-Rietschel, Fiona Dunlevy, Veerle Bulteel, Kate Hayes, Anne Verbrugge, Hettie M. Janssens, Nadine Dufeu, Nicholas J. Simmonds, Lieven J. Dupont, Damian G. Downey

**Affiliations:** 1 University Hospital Cologne, Faculty of Medicine, University of Cologne, Kerpenerstr. 62, 50937 Cologne, Germany; 2 European Cystic Fibrosis Society, Karup, Denmark; 3 Queen’s University Belfast, Belfast, UK; 4 Erasmus MC-Sophia Children’s Hospital, Rotterdam, The Netherlands; 5 University Aix-Marseille, Marseille, France; 6 Royal Brompton Hospital, London, UK; 7 University Hospital Leuven, Leuven, Belgium

**Keywords:** Pandemic, Mitigation, SARS-CoV-2, COVID-19, Clinical trials, Cystic fibrosis

## Abstract

The SARS-CoV-2 pandemic has disrupted clinical trials worldwide. The European Cystic Fibrosis Society-Clinical Trials Network (ECFS-CTN) has tracked clinical trial disruption by surveying its 58 trial sites across 17 European countries and collated information on measures to mitigate the impact of the pandemic and ensure trial continuity. Here, we present recommendations on how to reduce the risk of SARS-CoV-2 exposure to patients and trial staff by implementing remote trial visits where possible, using home assessments, video and phone calls, electronic consent, and home delivery of study drugs. We discuss the practicalities of remote source data verification, protocol amendments, changing trial site location, and staff absences and home working. We outline recommendations on how to protect trial outcomes, including home assessments, safety reporting, protocol deviations, and recruitment challenges. Finally, we discuss the importance of continued access to study drugs via extension trials for some patients. This guidance was co-created from the shared knowledge and experience of sites in our network and was re-distributed directly to all ECFS-CTN sites to help mitigate the impact of further waves of the SARS-CoV-2 pandemic. We will also use this guidance to assist companies, academia, and consortia with future protocol design and risk mitigation plans. This guidance can be applied to clinical trials in other diseases and could help sites that are not supported by clinical trial networks.

## Background

The severe acute respiratory syndrome coronavirus 2 (SARS-CoV-2) pandemic has disrupted the conduct and setup of clinical trials in diseases other than SARS-CoV-2 infection, largely due to mitigation efforts including self-isolation and reorganization of hospitals and staff to handle escalating admissions of infectious patients and diversion of resources towards clinical trials of treatments and vaccines for SARS-CoV-2 infection [1]. Data from ClinicalTrials.gov showed a marked decrease in the number of new clinical trials activated during February and May 2020 compared to previous periods [2]. Clinical trial teams, hospitals, sponsors, and competent authorities have scrambled to adapt to the challenges posed by the pandemic. A systematic review of articles describing the impact of the pandemic on clinical trials highlighted delayed enrollment and operational challenges in ongoing trials [3]. The overriding concern is the protection of clinical trial participants and healthcare professionals involved in trial conduct. The FDA issued regulatory guidance for sponsors in mid-March 2020 [4], closely followed by EMA guidance a few days later [5]. Guidance for ongoing trials includes switching to telephone/remote visits when possible, extending trial duration, home delivery of study drugs, and remote monitoring of trial conduct and source data by the sponsor. These documents also provide guidance on protocol amendments, updating informed consent in case of substantial protocol amendments, documenting protocol deviations, and handling missing data after trial completion. The regulators also advised sponsors to assess the feasibility of starting new trials and enrolling new patients into ongoing trials in light of the pandemic [4, 5].

Since 2009, the European Cystic Fibrosis Society-Clinical Trials Network (ECFS-CTN) has been working to improve the quality and conduct of industry-sponsored clinical trials in the rare disease cystic fibrosis (CF), with the aim of accelerating the availability of new therapies. We currently federate 58 trial sites across 17 countries in Europe. We do not act as a contract research organization (CRO), nor do we conduct clinical trials for commercial sponsors. Rather, our work includes a review of study protocols and investigator brochures, development and standardization of clinical trial measures and outcomes, training of investigators and research coordinators, and quality assurance of trial sites [6].

As SARS-CoV-2 spread across Europe in early 2020, it became clear that the crisis would impact the conduct of clinical trials in CF [1], especially since it was feared that people with CF would experience severe illness if infected with SARS-CoV-2 [7]. We started regularly surveying our member sites using an online survey tool to understand the impact on CF trials across Europe. The results of each survey were promptly returned to sites and partner patient organizations. We previously described descriptive quantitative survey results for spring 2020 [1], then up to the end of 2020 [8].

Here, we describe a follow-up collaborative qualitative project to identify the risks posed by the pandemic to the conduct of cystic fibrosis clinical trials and to create recommendations on how to mitigate these risks.

## Methodology

We used an online tool to survey our 58 sites eight times between March and December 2020, as previously described [1, 8]. Over the summer of 2020, we took advantage of the lull in SARS-CoV-2 infections to review the anecdotal free-text responses of the surveys up to June 2020. Thematic mapping and purposive sampling to identify risks and relevant mitigation measures were conducted by 5 individuals within the project team (SvK, VB, AV, FD, KH), according to published methodology [9–11].

These draft risks and recommended mitigation measures were complemented with experience from the ECFS-CTN coordinating center and then agreed by two iterative consensus rounds, first with the author group of this paper, then by the wider group of investigators and research coordinators from the 58 ECFS-CTN sites. Due to the time pressure necessitated by the pandemic, focus groups and/or interviews which could have further developed these themes were not deemed practical given the urgency of the situation and work-related pressure on respiratory-based site staff. This could form the basis of a future study, in line with the Equator Network guidelines.

## Risks and recommended mitigation measures

Our collaborative approach allowed us to source the best mitigation practices from CF trial sites all over Europe, thereby providing our member sites with useful guidance that could be locally adapted to mitigate the effects of the pandemic on clinical research and ensure continuity of existing and new trials for CF treatments.

We identified risks that could be impacted by resurgences of SARS-CoV-2 infection, or in many cases, by other emergency situations. Here, we present our recommendations for mitigating these risks (Table 1). These recommendations are not binding; rather, they provide sites with a list of items to consider and to potentially adapt to their local situation and institutional guidelines, along with any protocol amendments implemented by sponsors for specific studies.

**Table 1:** Risks to clinical trials in cystic fibrosis

Risk	How to mitigate the risk
**Preventing SARS-CoV-2 infection**	
*Infection risk prevents onsite visits* A physical visit at the site is not possible/inadvisable due to the risk of infection.	Where possible, replace trial visits with “remote” visits by telephone or video.
*Informed consent blocked by lack of onsite visits* Informed consent process may not be possible if patients are not allowed to attend trial visits in clinics.	Use electronic consent, if allowed by national and local regulations.
*Study drug dispensing blocked by lack of onsite visits* Study drugs cannot be provided if the patient is not allowed to come to the site.	Ship study drugs to patient’s home, if allowed by national and local regulations.
*Infection risk with necessary onsite trial visits* Patients could be infected or have been exposed to SARS-CoV-2.	Pre-screen patients by telephone for symptoms of infection or arrange a test before the trial visit.
**Logistics**	
*Monitors not allowed onsite for source data verification (SDV)* Unverified data could impact the robustness of the trial results.	Remote source data verification is allowed in some countries.
*Increased protocol amendments* Mitigation measures may lead to more protocol amendments than usual.	Update site’s workflow for handling protocol amendments, taking into account local situations and restrictions.
*Trial site may be moved to a different location* Reorganization of hospitals may mean that the trial site is effectively moved to another location within the hospital or even to another hospital.	Communicate relocation to patients, sponsors, local ethic committees and all other relevant partners. Adapt study material to the setting of the new location.
*Clinical trial staff unavailable* Clinical staff (investigators, nurses, etc.) may fall ill, have to be quarantined, and be reassigned to clinical duties or SARS-CoV-2 clinical trials. Non-clinical trial staff may be required to work from home, reducing access to vital documents.	Make sure to adequately train any replacement personnel on site- and protocol-specific procedures. Home working policies and effective information technology solutions are needed to cater for staff forced to work from home.
*Finances and other resources* Mitigation measures may elicit extra costs or extended timelines. The clinical trial supply line can also be disrupted by the pandemic.	Communicate early with commercial sponsors regarding extra costs and with non-commercial trial funders regarding grant extensions. Keep an up-to-date inventory of clinical trial materials and re-order early.
**Protecting trial outcomes**	
*Missed assessments* Assessments cannot be done at the site due to the risk of infection.	Prioritize assessments underpinning the primary endpoint. Use home health services and provide equipment for home measurements, where possible.
*Study drug compliance not checked* If patients are not coming to the site, compliance on returned study drug packaging cannot be verified.	Ask patients to save empty study drug packaging for return to the clinic later, or ask patients to transmit photographs of empty packaging.
*Increased adverse events* SARS-CoV-2 infection can lead to increased adverse events.	Check with protocol and sponsor how to handle such adverse events.
*Increased protocol deviations* Due to pandemic restrictions, there may be more protocol deviations.	Check with protocol and sponsor how to handle such protocol deviations.
*Recruitment and retention problems* Recruitment may be below expectations, expected patients drop out, or/and new patients are not allowed to come to the clinic.	Check whether the recruitment window can be extended. Remote assessments and trial visits may improve recruitment and retention.
**Continued access to study drugs**	
*Hospitals may block the initiation of new trials* Blanket bans on new trial initiation may block long-term extension trials following pivotal phase 3 trials. This can jeopardize continued access to study drugs for patients while waiting for authorization and reimbursement.	Investigators, learned societies, clinical trial networks, and patient organizations can advocate for these trials to be treated as high-priority trials.

### Preventing SARS-CoV-2 infection

Ensuring the safety of study participants and staff was key. Patients may be wary of coming to the clinic for trial visits and becoming infected. Conversely, they could be asymptomatic or pre-symptomatic transmitters of SARS-CoV-2 and infect the staff when they come to the clinic.

#### Infection risk prevents onsite visits

If local travel restrictions are in place, onsite trial visits can be minimized if they can be performed by telephone or video call using safe platforms. Some countries allow home visits by qualified personnel. Clinical trial visits could be scheduled and combined with routine clinical care visits to reduce the risk of infection if patients need to be seen at the trial site. Face-to-face site visits should only occur if they cannot be performed remotely. Sites should prioritize onsite visits that assess primary outcomes and important safety parameters, e.g., liver function tests. Patients attending onsite visits should be offered the possibility to come by private taxi if their only alternative is public transport. Sites and clinical trial networks should advocate that sponsors directly pay for these extended travel costs to avoid out-of-pocket expenses to trial participants.

#### Informed consent blocked by lack of onsite visits

If patients are not attending the clinic, it can be difficult to obtain informed consent for participation in new trials or to continue participation in existing trials following a protocol amendment. Electronic consent (eConsent) can be a solution. Several apps and platforms exist for electronic consent; however, regulations governing their use vary by country and even by institution. The use of eConsent platforms may need to be approved by local ethics committees. Sites should check if the study sponsors can offer technical solutions for eConsent.

#### Study drug dispensing blocked by lack of onsite visits

The shipment of study drugs directly to patients’ homes can also reduce clinic contact time. Again, not all national and local regulations allow this. Patients may need to be educated on the proper storage of study drugs and administration. Temperature requirements for the transport of study drugs must also be considered.

#### Infection risk with necessary onsite trial visits

If a patient must attend a trial visit in person, we recommend that they are pre-screened for symptoms of SARS-CoV-2 infection via email, telephone, or a video call. Alternatively, they could provide a recently negative SARS-CoV-2 PCR test result (< 48 h before the planned visit), especially if the trial visit involves aerosol-generating procedures. Self-administered rapid antigen diagnostic tests may be permitted in some countries and institutions, but PCR testing remains the gold standard.

### Logistics of trial conduct

Conducting clinical trials during a pandemic creates many logistical challenges. For example, external clinical trial monitors may not be allowed into hospitals to verify the source data. Clinical trial staff and infrastructure may become unavailable for clinical trials as hospitals reorganize to care for patients infected with SARS-CoV-2. Finally, the changes implemented can require protocol and budget amendments.

#### Monitors not allowed onsite for source data verification (SDV)

Many hospitals have suspended the possibility of external visitors such as site monitors [1], forcing sponsors to switch to remote source data verification (SDV). Sites in countries that allow remote SDV should work with their sponsor and CRO to determine what information can be shared and how, while remaining compliant with data protection regulations. We previously observed that remote SDV is more time-consuming than face-to-face SDV [1]. This could be dealt with by a budget amendment, with costs covered directly by the commercial sponsor. Proactive planning can help sites handle the increased workload of “catch-up” monitoring that will occur when restrictions are lifted. Site staff may require training on remote SDV procedures. A protocol amendment (and ethics committee approval) may be required to facilitate remote SDV.

#### Increased protocol amendments

Protocol amendments are likely for ongoing trials. If sites do not already have standard workflows for handling protocol amendments, they should plan workflows for gaining approval (from ethics committees, competent authorities, and local committees), training site staff, and re-consenting participants, if necessary.

#### Trial site may be moved to a different location

Some hospitals have been completely reorganized to separate SARS-CoV-2 and non-SARS-CoV-2 services. Changing the physical location of a trial site to another part of the same hospital, or even to a different hospital, can help reduce the risk of exposing trial participants to SARS-CoV-2, but requires significant preparation, both physical and administrative. Clear communication with the entire clinical trial team is critical, and the date of the relocation should be communicated to the patient, sponsor, and other relevant partners. Any equipment moved will need to be recalibrated. Fridges and freezers will need to be equipped with alarm systems for temperature monitoring. Access to time-sensitive consumables such as dry ice should be verified before any patient visits. Couriers should be notified of any change in address for sample pickup. If applicable, pharmacy staff should be involved while planning the move to check whether the study drugs can be distributed to the new location (if home delivery of study drugs has not already been established). The new trial location should be equipped with locked cabinets to protect patient and trial data, and any relocation of files, study material, storage conditions, changes in equipment, etc. should be documented. Any new personnel associated with the move should be trained appropriately and added to the delegation log. Finally, the local ethics committee should be notified of the change in location.

#### Clinical trial staff unavailable

Staffing levels can be reduced if clinical trial staff (research coordinators, nurses, and investigators) are reassigned to SARS-CoV-2 clinical duties or trials, fall ill, or have close contact with a confirmed case, leading to obligatory isolation at home [1].

If clinical research duties must be delegated to other team members, they should undergo the relevant training (good clinical practice [GCP], protocol-specific, etc.). All personnel changes should be documented in delegation and training logs. In some cases, the local ethics committee may need to be notified, particularly if the principal investigator changes.

Some trial staff, who have more administrative, regulatory, or data management roles, may be forced by institutional regulations to work from home to reduce exposure to the virus. Sites and their institutions should develop home working policies as a priority, with technical solutions such as providing home-based staff with encrypted computers and secure networks to access documents. Home working policies should provide staff with guidance on data protection.

#### Finances and other resources

The SARS-CoV-2 pandemic and the mitigation measures suggested in this paper invariably have a financial impact. We have focused on industry-sponsored trials here and advise sites to discuss the cost of mitigation measures upfront with sponsors. However, many trials are investigator-initiated and funded by academia or grants from charities, national research bodies, or European programs. We advise sites participating in such trials to immediately open communications with the trial funder to discuss extra funding for mitigating the impact of the pandemic or for grant extensions to account for delays caused by the pandemic.

The pandemic may also disrupt the clinical trial supply chain, creating a shortage of ancillary supplies for clinical trials. Sites should be aware of their inventory of clinical trial-related materials and should interact with sponsors and CROs to adequately forecast patient recruitment.

### Protecting trial outcomes

Pandemic-related disruption of ongoing trials could prevent the robust assessment of outcomes, in particular, safety and the primary outcome. It is critical to minimize trial disruption to preserve the integrity of ongoing trials and to avoid the delayed introduction of potentially life-extending medicines. This is particularly important for life-threatening rare diseases with limited treatment options such as CF. It is also an ethical obligation to ensure that trials avoid research waste by adequately answering the research question.

#### Missed assessments

Assessments underpinning the primary endpoint should be prioritized. If sponsors do not proactively provide guidance, sites can ask the sponsors to provide a minimal core data set (plan “B”) outlining which outcome measures can be obtained remotely where possible.

If patients are provided with the relevant sterile equipment and instructions (written, by telephone, or video), they can provide biological samples (e.g., urine, sputum) from home, which can be collected by the courier and transported to the hospital. Other common assessments that can be performed at home include filling in any patient-reported outcome questionnaires, pregnancy testing, height, and weight (ideally using equipment provided and calibrated by the sponsor). This will require close collaboration with the sponsor and consideration of extra costs. Ethics approval could be needed for such amendments.

Some assessments can be performed at home by private home care services hired by the sponsor, or by a site research nurse or coordinator in some cases. These include electrocardiogram, blood draws, vital signs, pulse oximetry, and spirometry (see case study in Table 2). Case report forms may need to be modified to account for such changes.

**Table 2:** Can spirometry performed at home be used as an outcome measure in cystic fibrosis trials?

Spirometry is a key assessment parameter in CF and is often a primary outcome in clinical trials. Patients require training to perform this maneuver, and normally, clinical trial staff oversee the test to ensure that the procedure is performed correctly—this is important to ensure the robustness of the results. With recent technological advances, compact home spirometry equipment is now available, but is not yet implemented as a standard in routine CF care. In addition, these devices have not yet been validated for the assessment of spirometry endpoints for clinical trials. In face-to-face spirometry assessments, patients are coached by the research nurse or coordinator regarding the necessary technique required to perform a “valid” forced expiratory maneuver. Spirometry performed at home, even when utilizing remote coaching via video-link, may therefore be qualitatively suboptimal as compared to an in-clinic assessment. This could lead to variable results, especially if some assessments are performed at home and others in the clinic. A study sponsored by the Cystic Fibrosis Foundation is investigating how home spirometry results can be “corrected” in order to be comparable to spirometry performed in clinics [12]. It is unknown at this point how regulators will evaluate the results of key outcome parameters obtained with unvalidated home equipment and this is an area requiring further research.	

#### Study drug compliance not checked

Checking compliance is an important way to ensure that patients are taking study drugs as instructed. This is often monitored by the patients returning empty study drug packaging to the trial site. If patients are not attending the clinic, they cannot bring back empty study drug packaging to assess compliance. Alternative ways to document compliance include having the patient photograph packages of used and unused study drug packaging (blister packs) and send these photographs to the site or check online during a video call and take pictures (screenshots) of patients demonstrating used and unused packages. Sites can also ask the patients to collect all packaging to return to the site when mitigation measures are no longer in place or can organize home collection. Sites should consult the study sponsor to choose the most appropriate method, considering any extra burden to be placed on patients and the potential need for ethical approval of the updated compliance methodology.

#### Increased adverse events

If trial participants become infected with SARS-CoV-2, there may be a rise in the number of adverse events (AEs) and serious adverse events (SAEs). Sites should discuss with the sponsor how to handle these AEs/SAEs. Patients should be reminded to call the trial team in case of any possible AEs. Phone/video calls can be performed to collect and record concomitant medications and AEs.

#### Increased protocol deviations

Similarly, restrictions related to the SARS-CoV-2 pandemic may lead to more protocol deviations, especially around missed visits and assessments. Sites should discuss how to handle and report protocol deviations related to the pandemic prior to database lock with sponsors.

#### Recruitment and retention problems

The pandemic may impact recruitment since patients may be more hesitant to enroll in new trials and may drop out of ongoing trials, or hospital rules may prevent patients from joining new trials. Remote trial visits and home assessments may increase acceptance of trial participation during a pandemic. Sites should discuss with the sponsor in advance under which circumstances the study will be stopped due to low recruitment or whether the recruitment window can be extended.

### Continued access to study drugs

Blanket bans on new trial initiation may block extension/rollover trials and continued access to investigational drugs. This is particularly important for patients for whom highly effective therapies do not exist or are not accessible. For the rare disease CF, we were concerned that this would block continued access to a new class of medicines called highly effective modulator therapy (HEMT) of the cystic fibrosis transmembrane conductance regulator (CFTR). For example, the triple therapy ivacaftor/tezacaftor/elexacaftor (Kaftrio, Vertex Pharmaceuticals) was approved by EMA in August 2020 [13] following unprecedented improvements in lung function and other key outcomes in clinical trials. Nevertheless, many people with CF accessed this medicine via long-term extension trials in 2020 and 2021 while waiting for EMA approval and then reimbursement negotiations in their countries. We previously reported that most ECFS-CTN sites were allowing extension trials of HEMT to start as planned, ensuring access to these study drugs [1].

Investigators, learned societies, clinical trial networks, and patient organizations can advocate for these trials to be treated as high-priority trials.

## Conclusions

These recommendations were created from the shared knowledge and experience of sites in our network and were subsequently distributed directly to all ECFS-CTN sites. We will also use this guidance to assist companies, academia, and consortia with future protocol design and risk mitigation plans. We will be able to monitor this via our protocol review and scientific advice service to sponsors wishing to run trials in ECFS-CTN sites. Indeed, many of the mitigation measures proposed in this manuscript are difficult to implement at the site level without the logistical and financial support of the study sponsor. At a network level, we can also promote the creation of a disaster management plan at the site level to plan for risks such as future pandemics, cyberattacks, or loss of infrastructure and/or key personnel.

Experience gained with virtual and remote technologies during the SARS-CoV-2 pandemic should be further explored by industry, regulators, CF clinical trial networks, investigative sites, and patient organizations to explore if such technology can enhance patient centricity in future clinical trials. Indeed, the rapid pivot towards home monitoring and other remote measures may pave the way for a future shift towards more “remote” trial visits.

Most of the learnings and recommendations we present in this article are not specific to CF and can be applied to clinical trials of drugs for any disease, whether rare or common. We hope that our manuscript assists trial sites that are not otherwise supported by a clinical trial network and that would benefit from the collective knowledge and experience of our ECFS-CTN member sites. Our advice for sites complements the recently published insights from another clinical trial network specialized in conducting nephrology investigator-initiated trials [14]. We also hope that our manuscript will serve as a starting point for continued conversation as well as sharing of experience to optimize best practice within the wider field of clinical trials.

## Data Availability

Not applicable.

## Abbreviations

AE: Adverse event
CRO: Contract research organization
CF: Cystic fibrosis
CFTR: Cystic fibrosis transmembrane conductance regulator
eConsent: Electronic consent
ECFS-CTN: European Cystic Fibrosis Society-Clinical Trials Network
GCP: Good clinical practice
HEMT: Highly effective modulator therapy
SAE: Serious adverse event
SARS-CoV-2: Severe acute respiratory syndrome coronavirus-2
SDV: Source data verification
